# Comparing Different Models of the Development of Verb Inflection in Early Child Spanish

**DOI:** 10.1371/journal.pone.0119613

**Published:** 2015-03-11

**Authors:** Javier Aguado-Orea, Julian M. Pine

**Affiliations:** 1 Department of Psychology, Sociology and Politics, Sheffield Hallam University, Sheffield, United Kingdom; 2 School of Psychology, University of Liverpool, Eleanor Rathbone Building, Bedford Street South, Liverpool, United Kingdom; University College London, UNITED KINGDOM

## Abstract

How children acquire knowledge of verb inflection is a long-standing question in language acquisition research. In the present study, we test the predictions of some current constructivist and generativist accounts of the development of verb inflection by focusing on data from two Spanish-speaking children between the ages of 2;0 and 2;6. The constructivist claim that children’s early knowledge of verb inflection is only partially productive is tested by comparing the average number of different inflections per verb in matched samples of child and adult speech. The generativist claim that children’s early use of verb inflection is essentially error-free is tested by investigating the rate at which the children made subject-verb agreement errors in different parts of the present tense paradigm. Our results show: 1) that, although even adults’ use of verb inflection in Spanish tends to look somewhat lexically restricted, both children’s use of verb inflection was significantly less flexible than that of their caregivers, and 2) that, although the rate at which the two children produced subject-verb agreement errors in their speech was very low, this overall error rate hid a consistent pattern of error in which error rates were substantially higher in low frequency than in high frequency contexts, and substantially higher for low frequency than for high frequency verbs. These results undermine the claim that children’s use of verb inflection is fully productive from the earliest observable stages, and are consistent with the constructivist claim that knowledge of verb inflection develops only gradually.

## Introduction

How children acquire knowledge of verb inflection is a long-standing question in language acquisition research (e.g.[1–11]). On the one hand, many constructivist proposals assume that children’s knowledge of verb inflection develops only gradually. For example, Tomasello [9] argues that children’s knowledge of verb inflection is initially tied to particular lexical items and only gradually becomes fully productive. On the other hand, many generativist proposals assume that children have productive knowledge of verb inflection from very early in development. For example, Wexler [10] argues that children know the inflectional properties of their language before they produce their first two-word combinations, and hence that children’s use of inflectional morphology is fully productive from the earliest observable stages.

These positions are obviously very different in principle. However, in practice, they are more difficult to distinguish than they might at first appear, particularly in a language like English, where the same zero-marked form of the verb can be used in a wide variety of different contexts. One way of distinguishing between them more clearly is to focus on data from more highly inflected languages like Spanish or Italian, which require the child to use one of a large number of different possible inflections every time a verb is produced. However, although there have been a number of studies of children learning Spanish and Italian, none of these studies have analysed children’s use of inflectional morphology in sufficient detail to distinguish clearly between the two positions. Constructivist analyses have tended to focus on the apparent lexical specificity of children’s early use of inflections. For example, Pizzuto and Caselli [12] report that children learning Italian initially use most of their verbs in only one form and take this as evidence that their knowledge of verb morphology is initially tied to particular lexical items. However, as Yang [13] points out, such lexically specific effects could be as much a reflection of the Zipfian [14] distribution of words in naturalistic speech as of limitations in the child’s underlying knowledge. For example, it could be that the reason why most of children’s verbs initially appear to be used in only one form is that most of the verbs used by the child occur so infrequently in the relevant speech samples that the chances of them occurring in more than one form are extremely low. In contrast, generativist analyses have tended to focus on the low overall frequency with which Spanish- and Italian-speaking children make inflectional errors. For example, Wexler [10] argues that the low frequency of subject-verb agreement errors in the data suggests that children’s use of inflectional morphology is fully productive from the earliest observable stages. However, as Rubino and Pine [15] point out, the error rates on which such arguments are based are potentially misleading since they collapse together information about inflectional contexts that occur with very different frequencies. This means that it is possible for children to show very low overall error rates despite showing much higher error rates for particular inflections than would be predicted by Wexler’s account.

In view of these problems, the aim of the present study is to conduct a stronger test of the predictions of some current constructivist and generativist accounts of the development of verb inflection. This will be done by focusing on a dense corpus of early child Spanish based on the spontaneous speech of two children: Juan and Lucía, recorded between the ages of approximately 2;0 and 2;6. The constructivist claim that children’s early knowledge of verb inflection is only partially productive will be tested by comparing the average number of different inflections per verb in matched samples of child and adult speech. The generativist claim that children’s early use of verb inflection is essentially error-free will be tested by investigating the rate at which the children made subject-verb agreement errors in different parts of the present tense paradigm. It should be noted at the outset that these analyses will not allow us to distinguish between all classes of constructivist and generativist models. This is because some generativist models assume a greater role for lexical learning than others. For example, Pinker’s paradigm building account [16] does not predict that children’s use of verb morphology will be fully productive from the earliest observable stages since it assumes that children construct morphological paradigms by abstracting over contrasting inflectional forms initially represented as unanalyzed wholes. However, they will allow us to test a class of generativist models that has been particularly influential over the past 20 years (e.g. [6,10,17,18]). These models assume that young children begin the process of morphological development with an abstract rule that checks the inflection of agreement-marked verb forms, and take the low frequency of subject-verb agreement errors in languages like Spanish and Italian as evidence in favour of this assumption (see [19] for an insightful discussion of the differences between Pinker’s paradigm building approach and more recent generativist approaches).

### 1.1. Constructivist Models of the Development of Verb Inflection

A central assumption underlying constructivist models of the development of verb inflection is that children’s knowledge of verb inflection develops gradually through a process of abstraction across instances in the input. This assumption is sometimes taken to predict that children’s early use of verb inflection will be completely unanalysed and hence that any evidence of early generalisation from one form to another represents counterevidence to a constructivist position (e.g. [13,17]). In fact, however, since constructivist models typically do not specify at what point in development the process of abstraction begins, what constructivist models really predict is that knowledge of verb inflection will develop gradually, and hence that children’s early knowledge of verb inflection will be only partially productive (i.e. will not generalise to all of the verbs in their vocabularies).

On the face of it, there appears to be a great deal of evidence that children’s early knowledge of inflection is only partially productive. Thus, studies of the acquisition of verb morphology in English indicate that there is a long period in which children fail to use appropriate tense and agreement morphology in a considerable proportion of obligatory contexts [3]. Moreover, studies of the development of verb morphology in more richly inflected languages such as Italian and Spanish suggest that children’s knowledge of verb inflection is initially tied to particular lexical items [12,20]. When taken together, these results suggest that children’s knowledge of verb inflection is considerably less productive than that of adults. However, there are several potential problems with this interpretation of the data.

The first problem is that it relies rather heavily on data from English-speaking children. However, because of its impoverished system of verb morphology, English is not a particularly good language for investigating the development of verb inflection. Thus, the vast majority of morphological errors in early child English involve the use of zero-marked verb forms in contexts that require a verb form that is overtly marked for tense and/or agreement (e.g. ‘Yesterday we go to the park’, ‘Dolly want milk’). Such errors have traditionally been treated as cases of inflection drop. However, in more recent generativist accounts, they are analysed as Root Infinitive (RI) errors (i.e. cases in which the child uses a zero-marked infinitive in a finite context). Since, under this interpretation, zero-marking errors are not agreement errors as such (i.e. reflect the use of a non-agreeing rather than an incorrectly agreeing verb form), the high frequency of such errors in early child English does not constitute evidence against the productivity of children’s early knowledge of inflection, and is perfectly consistent with the claim that children’s knowledge of inflection is fully productive from the earliest observable stages [21].

A second problem is that claims about the limited flexibility of children’s knowledge of verb morphology typically ignore sampling considerations—and hence the possibility that the lexical specificity of children’s early language use may be as much a reflection of the distributional properties of naturalistic speech samples as it is of the limited nature of children’s underlying knowledge. Thus, the idea that limitations in the flexibility of children’s use of a verb inflection reflect limitations in the productivity of their knowledge of verb inflection rests on the implicit assumption that competent speakers of the language make significantly more extensive use of the productive possibilities of the verb inflection system in their own spontaneous speech. In fact, however, this assumption is rather questionable since, given the lexical frequency statistics of natural languages, adults’ use of verb inflection, like children’s, is likely to be highly skewed towards high frequency words and inflections, such that most verbs are used so rarely that they are unlikely to occur in more than one form [13]. It follows that, before taking the low flexibility of young children’s use of verb inflection as evidence of a lack of productivity, it is necessary to show that children’s use of verb inflection is significantly less flexible than that of competent speakers subject to the same sampling limitations (e.g. by comparing the flexibility of children’s and adults’ use of verb inflection in matched speech samples).

A final problem is that, even to the extent that children’s language use is less flexible than that of adults, differences in the flexibility of children and adults’ language use are likely to be confounded with differences in lexical knowledge of particular inflections. Thus, generativist theories typically distinguish between abstract knowledge of inflection and lexical knowledge of the inflections of the particular language being acquired [6,10,22]. Moreover, since the inflections of particular languages clearly have to be learned, one way of explaining why young children use verbs with fewer different inflections than competent speakers is in terms of absence of knowledge of particular inflections. Indeed, Hyams [23] argues that the apparently gradual nature of the development of knowledge of inflection in Pizzuto and Caselli’s Italian data [12] could easily be explained in terms of the gradual accumulation of lexical knowledge (i.e. knowledge of verb stems and knowledge of verb inflections) and is hence irrelevant to the claim that children have abstract knowledge of inflection. The implication is that, in order to show that young children’s knowledge of verb inflection is less productive than that of competent speakers of the language, one would need to control not only for differences in sample size and vocabulary range, but also for differences in lexical knowledge of the relevant inflections (e.g. by restricting one’s analysis to a fixed set of inflections, all of which were available to both the adult and the child from the point at which the analysis began).

In view of the above problems, one of the aims of the present study is to test the constructivist claim that children's early knowledge of verb inflection is only partially productive while controlling for some of the potential confounds in previous research. This will be done by adopting a novel approach to the assessment of productivity in which the number of different inflections per verb is compared in matched samples of child and adult speech. It should be noted that this comparative approach to productivity differs from traditional criterion-based approaches to productivity in two important respects. First, it treats productivity as a continuous dimension rather than as a binary distinction. That is to say, rather than assuming that knowledge is either unanalysed or productive, it allows for the possibility that the child’s knowledge of particular inflections, and hence of the system as a whole, may be less than fully productive even when the child is able to use all of the inflections within the system productively to some extent. Second, it focuses on the question of how productive children’s knowledge is rather than the question of when children’s knowledge becomes productive. That is to say, it is designed to investigate whether children’s use of verb inflection is significantly less flexible than one would expect if the system were fully productive, rather than to establish when particular verb inflections become partially or fully productive.

This kind of approach is obviously particularly well suited to the task of testing constructivist models of development. However, it also allows for a more direct comparison of constructivist and generativist models of the development of verb inflection than has been possible hitherto. This is because it allows one to contrast the constructivist prediction that children’s early use of verb inflection will be less flexible than one would expect if their knowledge of the system were fully productive with the prediction implicit in generativist models that children’s use of verb inflection will be as flexible as that of competent speakers of the language once one has controlled for differences in vocabulary range, sample size and knowledge of particular inflections (see [24] for a similar approach to investigating the early productivity of syntactic categories).

### 1.2. Generativist Models of the Development of Verb Inflection

A central assumption underlying many recent generativist models of the development of verb inflection is that children’s early use of verb morphology reflects innately specified knowledge of agreement coupled with early knowledge of inflection. For example, Wexler [10] claims that ‘At the earliest observable stage (from the time that the child enters the two-word stage around 18 months of age) the child knows the grammatical and phonological properties of many important inflectional elements in their language” (page 25). This assumption results in two critical predictions about children’s early use of verb inflection. First, since knowledge of agreement does not have to be learned, children’s use of the inflections that they know should be fully productive from the earliest observable stages. In other words, although children may not know all of the verb inflections of their language, they should, in principle, be able to use all of the verb inflections that they do know with all of the verbs in their lexicon. Second, since the child’s knowledge of verb inflection is assumed to be adult-like from the beginning, children’s use of verb inflection should be essentially error-free. In other words, children should not make subject-verb agreement errors in their early speech (or at least only in cases where they do not yet know the target inflection).

In principle, this kind of generativist model could be tested by focusing on either of these predictions. However, in practice, generativist analyses have tended to focus on the second prediction on the assumption that children could not show error-free performance unless they had fully productive knowledge of the system in question. For example, both Wexler [10] and Hoekstra and Hyams [6] review data on the rate at which children make subject-verb agreement errors in a number of different languages including Italian ([12,25]) and Spanish ([26]). Since subject-verb agreement errors never occur at rates of more than 5%, they conclude that children have productive knowledge of verb inflection from the earliest observable stages.

On the face of it, the low rate of subject-verb agreement error in highly inflected languages would appear to provide strong evidence in favour of generativist models of the development of verb inflection. It is certainly difficult to see how a child with only partially productive knowledge of verb inflection could avoid making subject-verb agreement errors in these languages, since they require the child to use one of several different possible inflections every time a verb is produced. In fact, however, the error rates on which generativist claims about subject-verb agreement are based are subject to two methodological problems that make them difficult to interpret.

The first problem is that these error rates collapse together information about inflectional contexts that occur with very different frequencies. This means that it is possible for children to show very low overall error rates despite showing much higher error rates in low frequency contexts than would be predicted by generativist models. For example, Rubino and Pine [15] report data on a child learning Brazilian Portuguese (a Romance language similar to Spanish), in which the overall rate of subject-verb agreement error was only 3%. However, this figure collapsed together error rates of 0.5% in high frequency third person singular (3sg) contexts and 43.5% in low frequency third person plural (3pl) contexts.

The second problem is that these error rates collapse together information about high and low frequency verbs, and fail to control for the frequency with which different verbs are produced by the child. This means that different verbs contribute different numbers of tokens to the overall error rate, and hence that the overall error rate is much more sensitive to the level of error on high frequency than on low frequency verbs. The implication is that the low overall error rate may hide much higher error rates on low frequency verbs [27].

In view of these problems, the second aim of the present study is to test the claim that children's early use of verb inflection is essentially error-free by conducting a detailed analysis of the pattern of subject-verb agreement errors in early child Spanish. This will be done by investigating the rate at which the children made subject-verb agreement errors in different parts of the paradigm (e.g. in 3sg versus 3pl contexts), and with different verbs (i.e. with high frequency versus low frequency verbs).

This approach clearly allows for a much stronger test of the generativist claim that young children’s use of verb inflection is essentially error-free than has been conducted hitherto. However, it also allows for a more direct comparison of generativist and constructivist models of the development of verb inflection. This is because it allows one to contrast the generativist prediction that children’s early use of verb inflection will be essentially error-free with the prediction implicit in constructivist models that low overall error rates will hide pockets of high error in low frequency parts of the system that reflect systematic gaps in children’s partially productive knowledge. It is sometimes argued that modern constructivist models do not predict high error rates in children’s early language (e.g. [9]). However, this argument relies on the assumption that children are restricting their use of lexical items (particularly verbs) to the contexts in which they have heard them used in the input. This may be a reasonable assumption when analysing children’s use of verb argument structure, where young children do appear reluctant to use verbs in transitive structures that they have only heard used in intransitive structures [28,29]. However, it is much less plausible when analysing children’s use of verb inflection, where errors could only be avoided if the child were to restrict her use of the verb forms she knew to the inflectional contexts in which she had heard them. For example, a Spanish child who had only heard the verb *Querer (Want-INF)* used in second person singular (2sg) contexts could only avoid making errors by restricting her use of *Querer* to 2sg contexts. One reason to doubt that young children restrict their verb use in this way is that, although caregivers tend to produce more 2sg than 1sg forms in their child-directed speech, young children tend to produce more 1sg than 2sg forms [30]. We therefore assume that constructivist models do predict inflectional errors, but only in contexts that require children to produce verb forms that they have not fully learned, and which they cannot generate on the basis of their partially productive knowledge of the system (i.e. in low frequency contexts and on low frequency verb forms that share little similarity with the high frequency forms that the child has already learned). The critical difference between current generativist and constructivist models is thus that, whereas generativist models predict essentially error-free use of verb inflection from the beginning, constructivist models predict pockets of high error in low frequency parts of the system, particularly during the early stages.

To summarise, the aim of the present study is to conduct a strong test of the predictions of some current constructivist and generativist accounts of the development of verb inflection. The constructivist claim that children’s knowledge of verb inflection is only partially productive will be tested by comparing the average number of different inflections per verb in matched samples of adult and child speech. The generativist claim that children’s early use of verb inflection is essentially error-free will be tested by investigating the rate at which the children made subject-verb agreement errors in different contexts and with different verbs.

It should be noted that, when testing these predictions, focusing on Spanish has two obvious advantages over focusing on English. First, Spanish has a much richer system of verb inflection than English, in which each verb can occur with up to 40 different inflections (see [31] for a detailed description of Spanish verb morphology). More specifically, whereas English has only one overt present tense inflection (3sg -s), and hence often does not require the child to produce an overtly inflected verb form, Spanish has 6 different present tense inflections, one of which is required every time a verb is produced in the present tense (see Table 1 for an outline of the Spanish system of present tense verb inflection). This means that, in principle, it is easier to detect both a) lack of productivity and b) high rates of subject-verb agreement error in early child Spanish than it is in early child English.

**Table 1:** The Spanish system of present tense verb inflection.

	Conjugation 1	Conjugation 2	Conjugation 3
Infinitive	Mir-ar (to Look)	Com-er (to Eat)	Viv-ir (to Live)
First person singular	Mir-o	Com-o	Viv-o
Second person singular	Mir-as	Com-es	Viv-es
Third person singular	Mir-a	Com-e	Viv-e
First person plural	Mir-amos	Com-emos	Viv-imos
Second person plural	Mir-áis	Com-éis	Viv-ís
Third person plural	Mir-an	Com-en	Viv-en

Second, since, in contrast to English, all present tense forms in Spanish are readily distinguishable from the infinitive, differences between the child and the adult, or developmental changes in the child’s data, cannot be explained in terms of an RI stage in which the child uses zero-marked non-finite verb forms in finite contexts. In view of the difficulties that young children can have producing /*r*/ in word-final contexts, it might be thought that, in practice, 3sg present tense forms such as *mira* and infinitive forms such as *mirar* might be more difficult to distinguish than they appear on paper. In fact, however, this is not the case, since, in Spanish, the last syllable of infinitive forms is stressed, whereas the last syllable of 3sg forms is unstressed. The dropping of word-final /*r*/ from infinitives would therefore result in forms such as *mirá*, which would be readily distinguishable from 3sg forms such as *mira*. Verb forms such as *mirá* were not observed in the present corpus. It follows that, sampling considerations and lexical learning aside, there is no reason, from a generativist perspective, why young Spanish-speaking children’s use of verb inflection should appear any less flexible or any more error-prone than that of fully competent speakers. That is to say, once Spanish-speaking children have learned a particular set of verbs and a particular set of inflections, their use of those inflections with respect to those verbs should be a) as flexible and b) as free of errors as that of older children and adults.

## Method

The present study represents an attempt to conduct a detailed analysis of the flexibility and accuracy of the use of verb inflection in early child Spanish. This will be done by focusing on a dense corpus of early child Spanish based on the spontaneous speech of two children: Juan and Lucía. In the first part of the method section, we describe the properties of this corpus. In the second part of the method section we describe the methodology used to measure the flexibility and accuracy of the children’s use of verb inflection.

### 2.1. The Aguado-Orea and Pine corpus

The dataset used in the study is a corpus of early child Spanish collected by the first author as part of his PhD research at the University of Nottingham, UK [32]. This dataset consists of naturalistic samples of speech from two Spanish children and their parents living in the Madrid area recorded longitudinally over a period between the ages of approximately 2;0 and 2;6. This period was chosen because previous longitudinal studies had shown that Spanish children start producing verbs within two word sentences at around the age of 2;0 (e.g. [33]), and that the following six months are typically a period of considerable growth in inflectional complexity [34]. The Aguado-Orea and Pine corpus is now publically available as part of the CHILDES database [35].

#### 2.1.1. Participants

Participants in the study were recruited through announcements made in Spanish-speaking e-mail lists for professionals interested in paediatrics, educational psychology, linguistics, language pathology and related disciplines. Children were selected on the basis that their families spoke the dialect of Spanish used in the Madrid area. A questionnaire was also given to parents interested in the study to screen out particularly slow language learners. This questionnaire was adapted by the first author from the Mexican-Spanish version of the MacArthur Communicative Development Inventory [36], and was used to check that the early verb use of the children selected fell within the normal range. Two children were selected on this basis: Juan and Lucía. Juan was a first-born boy whose family lived in the Madrid area. He participated in the study between the ages of 1;10.21 and 2;5.29. Both of his parents were monolingual Spanish speakers with higher degrees. Lucía was a first-born girl whose family also lived in the Madrid area. She participated in the study between the ages of 2;2.25 and 2;7.15. Both of her parents were monolingual Spanish speakers with higher degrees.

Ethics Statement

This study was approved by the Research Ethics Committee at University of Nottingham. According to the guidelines of this committee, written consent was obtained from the main caregivers for both children participating in the study. All signed consent forms are kept by the first author.

#### 2.1.2. Recording and Transcription

The Aguado-Orea and Pine corpus consists of naturalistic data recorded by Juan and Lucía’s parents without the presence of an investigator. Sessions were video-recorded in the home using a digital camcorder with a small tripod. The parents were given some basic recording guidelines (e.g. avoid noisy backgrounds and try to record ‘varied’ and ‘interactive’ situations), but were told that the main aim of the study was to collect as large and as ‘natural’ a sample of their child’s spontaneous speech as possible. Juan and Lucía’s parents typically recorded 2 or 3 sessions per week, with each session lasting between 20 and 40 minutes (M = 29 minutes for Juan and M = 29 minutes for Lucía). The resulting corpora included 1930 minutes for Juan (61 minutes per week on average) and 1387 minutes for Lucía (68 minutes per week on average).

Recordings were transcribed according to the CHAT conventions of the CHILDES system [35]. An additional coding tier was also added to the corpus. This tier was similar to the standard %mor line, but included additional information about verb morphology (e.g. the conjugation of the verb and whether the verb suffix was regular or irregular). Children’s imitations and self-repetitions were labelled and excluded from the analyses, as were utterances containing unclear or unintelligible segments. Songs, stories and routines were also labelled and excluded.

#### 2.1.3. Properties of the Aguado-Orea and Pine corpus

An overview of the properties of the Aguado-Orea and Pine corpus is provided in Table 2. This table includes data on the total number of utterances provided by all six participants, the number of utterances with verbs, and the percentage of utterances that included verbs (in brackets). The corpus differs from other corpora of early child Spanish in two important ways (see [37] for a review of these corpora). First, the adult participants in the study were Juan and Lucía’s fathers rather than their mothers, although the children’s mothers were also frequently present when the recordings took place. This was simply because, for both children, the father was the primary caregiver, and was at home with the child throughout the day while the mother was out at work. Some studies have found differences in the characteristics of maternal and paternal speech to language learning children (see [38] for a review). However, given that adult data are only used in the present study to provide control measures against which to compare the child measures, there is no reason to believe that such differences had any impact on the results reported below.

**Table 2:** Properties of the Aguado-Orea and Pine Corpus.

Participant	Starts	Ends	Utterances	Utterances including a verb
Juan	1;10.21	2;5.29	15,945	5,273 (33.1%)
Juan’s Father			20,456	12,961 (63.4%)
Juan’s Mother			8,494	5,214 (61.4%)
Lucía	2;2.25	2;7.15	10,616	2,692 (25.4%)
Lucía’s Father			13,294	7,139 (51.3%)
Lucía’s Mother			6.613	2,973 (45.0%)

Second, and more importantly, because recordings were made very frequently over a relatively short period of time, the Aguado-Orea and Pine corpus is much denser than any previous corpus of early child Spanish. Thus, Juan and Lucía’s corpora not only contain 100% and 35% more child utterances, respectively, than the next largest corpus of early child Spanish (the López Ornat corpus), but were also recorded over a much shorter period of time (6 months as opposed to 28 months), with the result that they contain far more utterances from the relevant period of development (i.e. the first half of the third year). These corpora therefore allow us to conduct a much more powerful test of claims about the flexibility and accuracy of children’s early knowledge of verb inflection than any previous corpus of early child Spanish.

### 2.2. Productivity Analyses

The main aim of the productivity analyses was to test the constructivist claim that Spanish-speaking children’s early knowledge of verb inflection is only partially productive while controlling for a number of factors that could potentially explain the apparently limited flexibility of children’s early use of verb morphology. This was done by calculating the number of different inflections per verb and comparing this with the number of different inflections per verb in matched samples of adult speech using paired-sample t-tests. All of these analyses focused only on verb tokens that were used correctly in context (the only exception to this rule was over-regularisation errors involving one of the 6 present tense inflections; since such errors clearly reflect the attempt to inflect a verb with the appropriate suffix, they were treated in the same way as correct present indicative verb forms and included in the analysis). They also focused only on verb stems inflected with one of the 6 present tense indicative inflections. All other verb forms were excluded from the analysis. Thus, the minimum possible level of productivity for both children and adults was 1 inflection per verb and the maximum possible level of productivity was 6 inflections per verb.

Adult-child comparisons were made by comparing the number of different inflections per verb in the speech of each child and his or her father while controlling for differences in vocabulary range, sample size and knowledge of particular inflections. Vocabulary range was controlled by restricting the analysis to verb stems that occurred at least twice in both the child and the relevant adult’s speech samples. Sample size was controlled by randomly sampling from the adult data the same number of verb tokens as was present in the child data. Sampling was conducted *with replacement* to guard against the possibility that drawing instances from a progressively decreasing pool of items might boost the number of different forms in the adult samples, and the measures derived in this way were based on average scores for 25 iterations of the sampling procedure to ensure their reliability. Finally, since first person plural (1pl) and second person plural (2pl) forms did not occur until relatively late in the children’s corpora, knowledge of particular inflections was controlled by restricting the analysis to 1sg, 2sg, 3sg and 3pl inflections beginning at the point at which the child had produced all of the relevant 1sg, 2sg, 3sg and 3pl suffixes in his or her speech. Controls for sampling differences and knowledge of particular inflections were applied cumulatively (i.e. by first controlling for vocabulary range and sample size, and then controlling for knowledge of particular inflections, vocabulary range and sample size), so that in the most restricted case, child and adult measures were compared while controlling for vocabulary range, sample size and knowledge of particular inflections all at the same time.

### 2.3. Error Analyses

The main aim of the error analyses was to conduct a strong test of the generativist claim that Spanish-speaking children’s early use of verb inflection is essentially error-free by calculating error rates separately for different parts of the verb system. Error analyses were restricted to children’s use of verb forms in present tense contexts and error rates were calculated in terms of the context to be filled as opposed to the form used (though see below). For example, the 1sg error rate was calculated as the percentage of 1sg contexts that were filled with a non-1sg verb form. The verb *Ser (to Be)* was excluded from the analysis on the grounds that, because it is highly irregular, any errors involving this verb would be difficult to interpret since they could simply reflect ignorance of the appropriate irregular form (in practice the patterning of errors with *Ser* was similar to that with other verbs, with by far the most common error being the use of the third person singular form in third person plural contexts).

#### 2.3.1. Inflectional errors by person and number

Analyses of inflectional errors by person and number were conducted by breaking down the present tense contexts produced by the child into 1sg, 2sg and 3sg, and 1pl 2pl and 3pl contexts, and calculating separate error rates in each case. Since Spanish is a null subject language, it is not always possible to identify the intended subject of the verb. Errors were therefore only coded when the person and number of the intended subject could be clearly identified on the basis of the discourse context. In order to control for the possibility that it might be easier to recover person and number information (and hence to identify errors) in some contexts (e.g. 3pl) than in others (e.g. 3sg), the analysis of the full dataset was followed up by an analysis restricted to verbs with overt subjects. For both children, the analyses based on the full and the restricted datasets revealed very similar patterns of results. Reliability was also assessed by having a second independent rater code all of the verb tokens in 10% of each of the children’s transcripts for both the target context and the identity of the inflection produced. Inter-rater reliability expressed as percentage agreement was 99% (*kappa* = 0.99)

#### 2.3.2. Inflectional errors by verb frequency

Analyses of inflectional errors by verb frequency were conducted by identifying the three verbs that occurred most frequently for each child in first person singular, second person singular and 3pl contexts, and calculating separate error rates for these verbs, and for the remaining verbs in each child’s corpus. The rationale for focusing on the three most frequent verbs in each case was that, since Lucía only used three verbs more than once in 3pl contexts, three was the highest number that could be used that would still allow a comparison to be made in all three cells for each of the two children.

#### 2.3.3. Inflectional errors by form produced

A final analysis considered which form of the verb the children used in those inflectional errors that they did produce. There is some suggestion in the previous literature that the majority of agreement errors that occur in early child Spanish involve the incorrect use of the 3sg present tense form in non-3sg contexts (e.g. [39,40]). This finding has been interpreted by some generativists as evidence that the 3sg functions as a non-finite form (or RI analogue) for the Spanish-speaking child (e.g. [41]). However, the 3sg is not only the most frequent form in both child and adult Spanish, but also the most phonologically prototypical form in the present tense paradigm (in the sense that it consists of the verb stem plus a thematic vowel, the identity of which is determined by the particular conjugation to which the verb belongs: -a for -ar verbs and -e for –er and –ir verbs). Children’s tendency to overuse 3sg forms is therefore also consistent with the constructivist hypothesis that children default to the most strongly represented form of the verb when they are unable to generate the correct form for a particular context.

## Results

The results of the present study are divided into two sections. In the first section we present a series of productivity analyses designed to test the constructivist claim that children’s knowledge of present tense verb inflection is less than fully productive. In the second section, we present a series of error analyses designed to test the generativist claim that children’s early use of verb inflection is essentially error-free.

### 3.1. Productivity Analyses

Table 3 presents data on the use of present tense inflections by Juan and Lucía and their fathers in the Aguado-Orea and Pine corpus. The upper part of Table 3 (Analysis A) includes data on all 6 present tense inflections (1sg, 2sg, 3sg, 1pl, 2pl and 3pl). These data provide an index of the extent to which the children and their caregivers made use of the productive possibilities of the Spanish system of present tense verb inflection in their interactions over the course of the study.

**Table 3:** Average number of different inflections per verb in the speech of Juan and Lucía and their Caregivers.

Participant	Inflections/verb	Tokens	Types	Verbs in only one form
Analysis A				
Juan	1.84 (1.15)	3014	144	77 (53.5%)
Juan’s Father	2.15 (1.42)	8300	268	128 (47.8%)
Lucía	1.64 (0.95)	1600	72	45 (62.5%)
Lucía’s Father	2.14 (1.41)	4386	174	86 (49.4%)
Analysis B				
Juan	2.27 (1.21)	2982	93	28 (30.1%)
Juan’s Father	2.64 (1.37)	2982	93	20 (21.5%)
Lucía	1.95 (1.02)	1540	43	19 (44.2%)
Lucía’s Father	2.36 (1.12)	1540	43	10 (23.3%)
Analysis C				
Juan	2.11	2544	81	23 (28.4%)
Juan’s Father	2.31	2544	81	21 (25.9%)
Lucía	1.79	859	35	15 (42.9%)
Lucía’s Father	2.11	859	35	9 (25.7%)

It can be seen from these data that, although, in principle, each Spanish verb can be used with 6 different present tense inflections, the average number of inflections with which verbs are actually used by the two children is closer to 1 than it is to 6 (1.84 for Juan and 1.64 for Lucía), with both children using more than half of the verbs they produced with only one inflection (53.5% for Juan and 62.5% for Lucía). These data could be taken as evidence that Spanish children’s use of verb inflection is less than fully productive during the early stages. However, it is clear from Table 3 that the adult figures are also closer to 1 than they are to 6, with both adults using around half of their verbs with only one inflection, and neither of them producing verbs with an average of more than 2.15 different inflections. This pattern of results is consistent with the claim that the lexically-specific look of children’s early use of verb inflection is at least partly a result of the Zipfian distribution of verbs in naturalistic speech [13], and underlines the need to consider the flexibility with which adults use verb morphology in their spontaneous interactions before taking the low flexibility of young children’s usage as evidence of a lack of productivity.

The middle part of Table 3 (Analysis B) presents data on the number of inflections per verb in the children and caregivers’ speech controlled for vocabulary range and sample size. It can be seen from these data that the vocabulary range and sample size controls (particularly the decision to restrict the analysis to verbs for which there were at least two tokens in both the child and adult samples) have a substantial effect on the percentage of verbs that occur in only one form in both the child and adult data (resulting in a reduction of approximately 20% in each case). This pattern of results is consistent with the idea that the very high percentages reported in Analysis A are a reflection of the Zipfian distribution of verb forms in the child and adult data. However, it is also clear from Analysis B, that the vocabulary and sample size controls have little effect on the difference between the child and adult measures, with the child measures remaining substantially lower than those of their respective caregivers (M = 2.27 versus M = 2.64 for Juan and M = 1.95 versus M = 2.36 for Lucía). These differences were analysed using paired-sample t-tests, which revealed that both Juan and Lucía produced significantly fewer inflections per verb than their caregivers (*t*_92_ = 4.48, *p*<.001, two-tailed for Juan and his Caregiver; and *t*_42_ = 3.15, *p* = .003, two-tailed for Lucía and her Caregiver). The implication is that the apparently limited productivity of Juan and Lucía’s use of verb inflection cannot be explained entirely in terms of the Zipfian distribution of verbs in naturalistic speech, and that both children’s knowledge of verb inflection is less than fully productive during the early stages.

The results presented so far are consistent with the constructivist claim that children do not have adult-like knowledge of verb inflection during the early stages. However, an alternative way of explaining the difference between the children and their caregivers that is consistent with a generativist position is in terms of differences in children and adults’ lexical knowledge of particular present tense inflections. The inflections of any particular language clearly have to be learned and, although some generativists (e.g. [10]) claim that children have mastered all the basic inflections of their language before they start to produce multi-word speech, it is obviously also possible that there is a stage in children’s early production when they are operating with only a subset of these basic inflections. The apparently limited nature of children’s early use of verb morphology may thus reflect ignorance of one or more of the inflections of the present tense system rather than the limited productivity of those inflections that the children are actually using. That is to say, it is possible that, although young Spanish-speaking children have yet to learn some of the inflections of the present tense paradigm, their use of those inflections that they have learned is nevertheless fully productive from the outset.

An obvious way of investigating this possibility is to restrict the analysis to a period beginning at the point at which the relevant child has already been shown to use all of a particular set of inflections in his or her speech. The lower part of Table 3 (Analysis C) presents the results of such an analysis (again controlled for vocabulary range and sample size). Note that this analysis is based on only 4 of the 6 possible present tense inflections (i.e. 1sg, 2sg, 3sg and 3pl), since the remaining inflections (i.e. 1pl and 2pl) did not appear until relatively late in both children’s corpora, which explains why the means for both the child and adult measures are lower than the means reported in Analysis B.

It is clear from these data that, despite the more targeted nature of the analysis, there is still a difference between the number of inflections per verb in the speech of both children and their respective caregivers (M = 2.11 versus M = 2.31 for Juan and M = 1.79 versus M = 2.11 for Lucía) These differences were analysed using paired sample t-tests, which showed that both Juan and Lucía produced significantly fewer inflections per verb than their caregivers (*t*_80_ = 2.84, *p* = .006, two-tailed for Juan and his Caregiver; and *t*_*34*_ = 2.61, *p* = .013, two-tailed for Lucía and her Caregiver). The implication is that the apparently limited productivity of Juan and Lucía’s use of verb inflection cannot be explained in terms of a lack of lexical knowledge of one or more of the relevant inflections, and hence that these children’s knowledge, even of those inflections that they are actually using in their spontaneous speech, is less than fully productive.

### 3.2. Error Analyses

Table 4 presents data on the rate of subject-verb agreement errors in present tense contexts in Juan and Lucía’s speech broken down by person and number. In each case, errors were coded as incorrect attempts to fill a (present tense) context. For example, a 1sg error was an error involving the use of a non-1sg form in a 1sg present tense context.

**Table 4:** Inflectional Errors by Person and Number.

Child	Context	Total	Error	%
Juan	1sg	665	31	4.7
	2sg	147	16	10.9
	3sg	1606	11	0.7
	1pl	61	0	0
	2pl	3	1	33.3
	3pl	186	63	33.9
	Total	2668	122	4.6
Lucía	1sg	489	15	3.1
	2sg	94	20	21.3
	3sg	671	2	0.7
	1pl	14	0	0
	2pl	0	NA	NA
	3pl	28	13	46.4
	Total	1296	50	3.9

It can be seen from Table 4 that, consistent with the findings of previous generativist work in this area, the overall rate of subject-verb agreement errors is very low (less than 5% for both children). However, it can also be seen that this low overall error rate hides very different error rates in different parts of the system. Indeed, it appears to reflect an interaction between differences in the rate at which errors occur in different contexts (<1% for 3sg versus 30 to 50% for 3pl contexts), and the highly skewed distribution of contexts in the children’s speech (>50% of the data for 3sg contexts versus < 10% of the data for 3pl contexts). These results show that the generativist claim that children’s use of subject-verb agreement is essentially error-free is empirically false, and reflects a failure to analyse the data at a sufficient level of detail. They also reveal a consistent pattern of error rates across the two children (where 3pl > 2sg > 1sg > 3sg). This pattern was analysed statistically by comparing each of these 4 error rates with each other using Chi-square (and Fisher’s Exact tests, where one or more of the expected values was less than 5). All of these comparisons were statistically significant for both children (all ps < .02, two-tailed).

The consistency in the pattern of error rates across the two children suggests real differences in the children’s knowledge of different parts of the present tense paradigm. However, one possible objection to this conclusion is that, because Spanish is a null subject language in which the subject of the verb is often left unexpressed, it might be easier to identify errors in some parts of the system than others—and that it might be this factor rather than differences in the children’s underlying knowledge that was driving the consistent pattern of error across the paradigm. In order to investigate this issue, an additional analysis was run on a subset of the data restricted to utterances with overt subjects. The results of this analysis are presented in Table 5, from which it can be seen that the pattern of errors in this more restricted dataset is very similar to that in the data for the children as a whole. Indeed, if one focuses on the error rates for 1sg, 2sg, 3s and 3pl, which are the only contexts that occur sufficiently often with overt subjects in either child’s data, there is a very high rank order correlation between the error rates presented in Table 4 and the error rates presented in Table 5 (*R*_*s*_ = 0.97, *N* = 8, *p*<.001, two-tailed). The implication is that the profile of errors across the different cells of the present tense paradigm is a real phenomenon that reflects differences in the children’s mastery of different parts of the system.

**Table 5:** Inflectional Errors in Utterances with Overt Subjects by Person and Number.

Child	Context	Total	Error	%
Juan	1sg	138	4	2.9
	2sg	24	6	25.0
	3sg	505	3	0.6
	1pl	1	0	0
	2pl	0	NA	NA
	3pl	102	39	38.2
	Total	770	52	6.8
Lucía	1sg	81	1	1.2
	2sg	27	3	11.1
	3sg	109	0	0
	1pl	0	NA	NA
	2pl	0	NA	NA
	3pl	12	6	50.0
	Total	229	10	4.4

One obvious explanation of this pattern of errors is that the rate at which children achieve mastery of the relevant inflections is related to the frequency of those inflections in the input. Thus, of the four contexts for which it is possible to calculate reliable error rates, 3sg is by far the most frequent and 3pl is the least frequent. However, it is important to note that although 1sg contexts are much more frequent than 2sg contexts in the child’s speech, they are actually less frequent than 2sg contexts in the input. The higher error rate in 2sg than 1sg contexts therefore cannot be explained in terms of input frequency.

The error rates reported in Tables 4 and 5 clearly suggest a more differentiated pattern of error than that predicted by current generativist accounts. They are also broadly consistent with the view that the rate at which errors occur is negatively related to the frequency with which different contexts occur in the input. However, it is important to recognise that even these error rates have the potential to hide more subtle patterns of error by collapsing together data from high and low frequency verb forms. We have already seen how the Zipfian distribution of verbs in naturalistic speech tends to exaggerate the lexically-specific nature of children’s early use of inflection. However, another consequence of the Zipfian distribution of lexical items is that it means that error rates that collapse across different verbs are likely to be particularly sensitive to the influence of high frequency items. This effect has the potential to hide pockets of high error with respect to low frequency items, particularly if high frequency items are more likely to be used correctly (e.g. because they occur in over-learned contexts).

**Fig 1:**
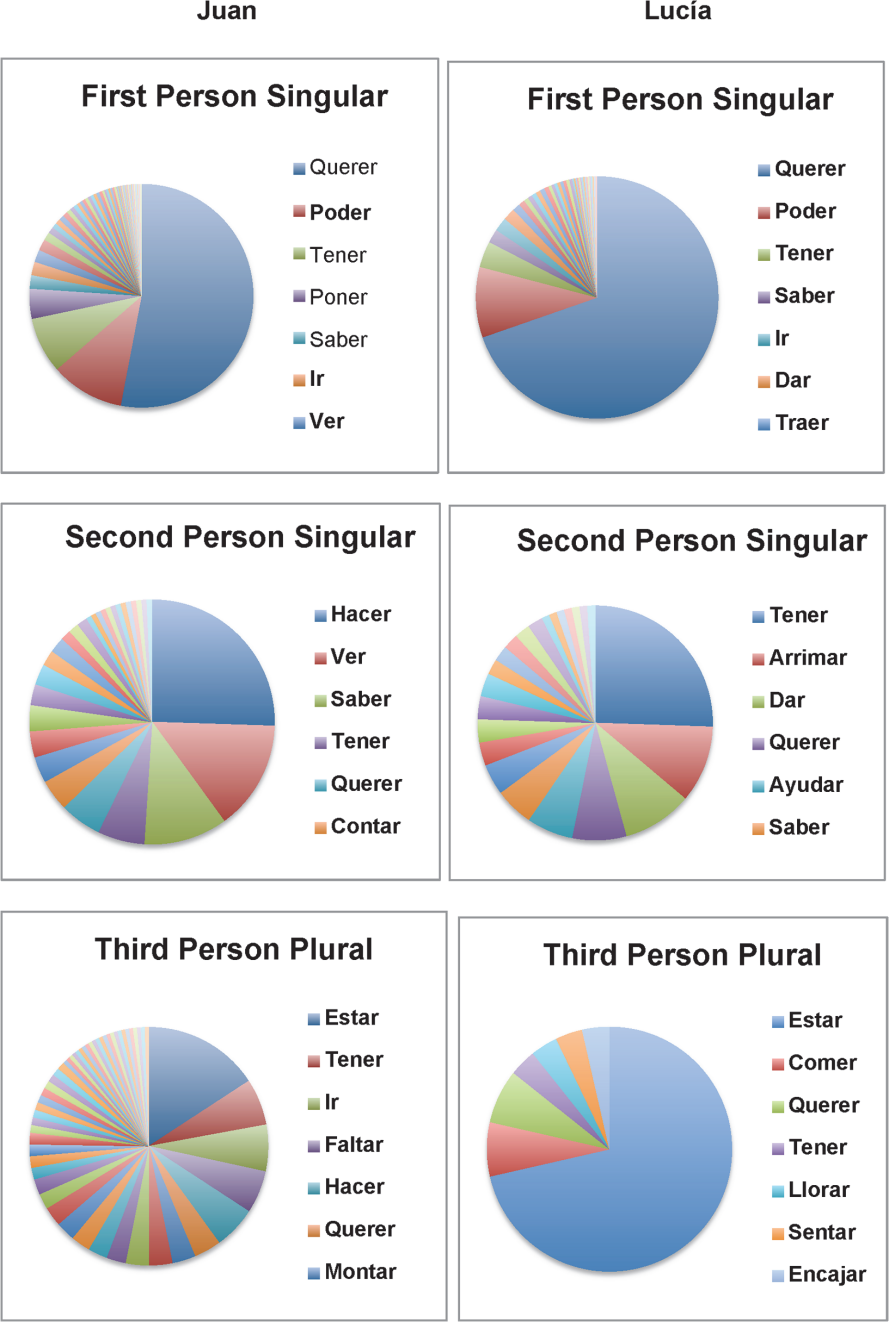
Distribution of first person singular, second person singular and third person plural contexts by lexical item in Juan and Lucía’s speech

Fig. 1 shows how Juan and Lucía’s production of 1sg, 2sg and 3pl contexts is distributed with respect to particular lexical verbs. It is clear from Fig. 1 that, in all cases, a relatively small number of high frequency verb forms make up a relatively large proportion of the data. This is particularly true for 1sg forms, where, for both Juan and Lucía, a single form: *Quiero (Want-1sg)* accounts for over 50% of 1sg contexts.

Table 6 shows how the distributions presented in Fig. 1 impact on error rates, by contrasting error rates based on the three highest frequency verbs in each cell and error rates based on the remaining verbs in each cell. Although the size of the differences varies considerably across cells, in all 6 cases, the error rates based on the three highest frequency verbs are lower than the error rates based on the remaining verbs (a difference which is significant by a Sign Test at *p* = 0.03, two-tailed). More importantly, it is clear that the children’s strong performance with respect to a handful of high frequency verbs often hides much weaker performance with respect to lower frequency verbs. Moreover, this is true not only for contexts in which the overall error rate is high (e.g. 3pl), but also for contexts which, in the previous analysis, appeared to be largely error-free (e.g. 1sg). These findings highlight another way in which the uneven distribution of contexts in naturalistic speech can disguise weaknesses in children’s knowledge of verb inflection, and provide further evidence against the claim that young children already have adult-like knowledge of verb inflection.

**Table 6:** Inflectional Errors by Frequency.

Child	Context	High Frequency			Low Frequency		
		Total	Error	%	Total	Error	%
Juan	1sg	479	8	1.7	186	23	12.4
	2sg	74	5	6.8	73	11	15.1
	3pl	56	17	30.4	130	46	35.4
	Total	609	30	4.9	389	80	20.6
Lucía	1sg	403	5	1.2	86	10	11.6
	2sg	42	8	19.0	52	12	23.1
	3pl	24	10	41.7	4	3	75.0
	Total	469	23	4.9	142	25	17.6

All of the error analyses presented so far have focused on how the children’s errors are distributed across different present tense contexts. However, a final question that is also relevant to our understanding of the children’s use of verb inflection is the question of which form of the verb the children tend to produce when they make an agreement error. An analysis of the errors reported in Table 4 revealed that the vast majority of both of the children’s errors (83.6% in the case of Juan and 92.0% in the case of Lucía) involved the use of a 3sg form. This finding is consistent with the previous literature on early child Spanish. It is also consistent with the idea that the children are defaulting to the most strongly represented form of the verb when they are unable to generate the correct form for a particular context.

## Discussion

The aim of the present study was to evaluate some current constructivist and generativist accounts of the development of verb inflection by focusing on data from a rich corpus of early child Spanish. The former were evaluated by testing the constructivist claim that children’s early use of verb inflection is less than fully productive. The latter were evaluated by testing the generativist claim that children’s use of subject-verb agreement is essentially error-free.

Our analyses of the productivity of children early use of verb inflection showed that, although even adults’ use of verb inflection in Spanish tended to look somewhat lexically restricted, children’s use of verb inflection was significantly less flexible than that of their caregivers. This effect was found even after controlling for verb vocabulary, sample size and knowledge of the relevant inflections, and hence cannot be explained in terms of sampling considerations or differences in lexical knowledge.

Our error analyses showed that, although the rate at which the two children produced subject-verb agreement errors in their speech was very low, this overall error rate hid a consistent pattern of error in which error rates were substantially higher in low frequency than in high frequency contexts, and substantially higher for low frequency than for high frequency verbs. These results undermine the claim that young children’s early use of subject-verb agreement is essentially error-free, and are consistent with the prediction implicit in constructivist models that low overall error rates will hide pockets of high error in low frequency parts of the system that reflect systematic gaps in children’s partially productive knowledge. They thus provide converging evidence in favour of the view that the children’s early knowledge of verb inflection is less than fully productive.

Of course, it is important to acknowledge that these results are based on data from only two Spanish-speaking children, and hence may not generalise to the all Spanish-speaking two-year-olds. On the other hand, they are remarkably consistent across the two children, with both children showing the same deficit in productivity relative to their caregiver, the same pattern of differences in error rate across the present tense paradigm, and the same tendency to produce errors involving the incorrect use of 3sg forms. When taken together, these findings have a number of implications for the field as a whole. First, they underline the need to take account of the distributional properties of naturalistic speech samples when investigating the productivity and accuracy of children’s early use of verb inflection. Thus, as Yang points out [13], an important feature of naturalistic speech samples is that they tend to conform to a Zipfian frequency distribution, where frequency decreases sharply as a function of rank order, such that a small number of items occur with relatively high frequency, but the vast majority occur with very low frequency. This feature of naturalistic speech has obvious implications for previous constructivist analyses of the development of verb inflection (e.g.[12,20]), which have tended to emphasise the fact that many of the verbs in children’s early speech samples occur with only one of a range of possible inflections. This is because many of the verbs in naturalistic speech samples occur so infrequently that even adult caregivers fail to produce them with more than one inflection. The implication is that measures of the flexibility of children’s use of verb inflection can only be meaningfully interpreted with reference to some kind of baseline measure of the level of flexibility that would be expected from a fully competent speaker. Interestingly, however, it also has implications for previous generativist analyses, which have tended to emphasise the very low rate at which inflectional errors occur. This is because the frequency with which particular inflectional contexts and particular verbs occur in children’s speech is so skewed that the overall rate at which inflectional errors occur tells us very little about the accuracy of children’s use of most of the inflections in the paradigm, or of most of the verbs in their vocabularies. The implication is that error analyses can only be meaningfully interpreted if they provide detailed breakdowns of children’s use of verb inflection by context and lexical item.

Second, our results suggest that, provided one collects sufficiently detailed data from a sufficiently targeted period of development, naturalistic data can be a very useful source of information about children’s early knowledge of inflection, at least in relatively highly inflected languages such as Spanish and Italian. Thus, the data collected in the present study were not only rich enough to allow us to measure differences in the flexibility of children’s and adults’ use of verb inflection, but also to identify reliable pockets of error (i.e. pockets of high error based on a relatively large number of relevant contexts), and reliable patterns of error (i.e. patterns of error that were consistent both across children and across utterances with and without overt subjects). These patterns clearly have the potential to inform models of the development of verb inflection, not only by highlighting the effect of factors such as frequency on the accuracy of children’s use of verb inflection, but also by identifying areas of weakness in children’s knowledge of the verb paradigm, which could be used to examine the role of other potential factors in the future. For example, one of the predictions implicit in current constructivist approaches is that children’s early knowledge of verb inflection will generalise probabilistically to verbs that the child has not heard inflected in a particular way on the basis of their similarity to forms that the child has heard inflected in that way. One way of testing this hypothesis would be to focus on 3pl contexts (where the error rate is high), and attempt to elicit low frequency forms that vary in terms of their semantic and/or phonological similarity to forms that the child knows. The prediction would be that children will produce fewer errors on low frequency verbs that are phonologically and/or semantically similar to 3pl forms that they know than they will on low frequency verbs that are phonologically and/or semantically dissimilar to such forms.

Third, our results suggest that, even in a language like Spanish, where the present tense paradigm is mastered relatively quickly, the development of verb inflection is a gradual process (i.e. a process that reflects probabilistic learning and generalisation rather than the instantaneous mapping of particular inflections to particular cells in a pre-given paradigm). This conclusion is clearly consistent with constructivist models of the development of verb inflection. The challenge for such models is to provide explanations of the pattern of productivity and error in young children’s speech that can be tested in future research. For example, one interesting feature of the subject-verb agreement errors identified in the present study is the fact that the vast majority of these errors (83.6% in the case of Juan and 92.0% in the case of Lucía) involved the use of a 3sg form in a non-3sg context. 3sg forms are not only the most frequent forms in both child and adult Spanish, but also the most phonologically prototypical forms in the present tense paradigm. This pattern of results can therefore be explained relatively easily on the basis that the children are defaulting to the highest frequency and/or most phonologically prototypical form in the input when they are unable to generate the correct form for a particular context (see [42] for evidence of a similar process operating in early child English).

A second interesting feature of the pattern of errors is the fact that both children made more errors in 2sg contexts than in 1sg contexts, despite the fact that the latter, although more common in the children’s speech, were less common in the speech of their caregivers. This effect is more difficult to explain in input-driven terms. However, one possibility is that 1sg contexts are protected from error in Spanish by the phonological distinctiveness of the target form (which does not contain a thematic vowel and hence is inflected in the same way across different verb conjugations). Another is that, despite their relatively low frequency in the input, 1sg contexts are particularly salient to language-learning children for pragmatic reasons, with the result that children learn and generalise across 1sg forms more readily than one would predict on the basis of input frequency alone (see [30] for a similar explanation of the pattern of auxiliary omission in early child English). Which of these explanations provides a better fit to the data is obviously a question for future research.

Finally, it is worth noting that, although consistent with constructivist models of the development of verb inflection, our results are not necessarily inconsistent with generativist approaches, provided that these approaches incorporate a role for probabilistic learning. For example, one generativist account that is consistent with the results of the present study is Pinker’s paradigm-building model [16]. This account is consistent with generativist theory, since it depends on the interaction between innate principles, such as the Unique Entry Principle, and exposure to contrasting inflectional forms in the input. However, since these contrasting forms are initially represented as unanalysed wholes, it also has the potential to explain the probabilistic nature of children’s early patterns of generalisation. Pinker’s account can be contrasted with an alternative possibility that is also consistent with generativist theory, which is the idea that the 3sg form is a default form, which is used by Spanish-speaking children when Agreement fails or is absent from the underlying representation of the sentence (e.g. [40,41]). Although this kind of account has the advantage that it is able to explain why children’s errors tend to involve the incorrect use of 3sg forms, it does not include a role for probabilistic learning, and so has no ready explanation for the consistent pattern of differences in the rate at which such errors occur across the present tense paradigm.

To summarise, in the present study, we have presented analyses of both the productivity and accuracy of two Spanish-speaking children’s use of verb inflection, which show that the claim that children’s knowledge of verb inflection is fully productive from the earliest observable stages is empirically false. Future work in this area, whether generativist or constructivist, should therefore focus on explaining developmental changes in the productivity and accuracy of children’s use of verb inflection rather than assuming that adult-like knowledge of verb inflection is already in place. One way of doing this is to collect naturalistic corpora that are sufficiently rich to allow the kind of detailed analyses reported here. Another is to use elicitation techniques to ‘zoom in’ on areas of weakness in the child’s use of verb inflection (which tend to be under-represented in naturalistic corpora because of their low frequency), and use the resulting pattern of errors to make inferences about the nature of the learning process.

## Supporting Information

S1 File Dataset files used in this study. (ZIP)

## References

[1] Berko J (1958) The Child's Learning of English Morphology. Word 14: 150–177.

[2] Bloom L, Lifter K, Hafitz J (1980) Semantics of verbs and the development of verb inflection in child language. Language 56: 386–412.

[3] Brown R (1973) A First Language The Early Stages. London: George Allen & Unwin Ltd. 437 p.

[4] Cazden CB (1968) The acquisition of noun and verb inflections. Child Development 39: 433–448. 5649958

[5] de Villiers JG, de Villiers PA (1973) A cross-sectional study of the acquisition of grammatical morphemes. Journal of Psycholinguistic Research 2: 267–278. 10.1007/BF01067106 24197869

[6] Hoekstra T, Hyams N (1998) Aspects of Root Infinitives. Lingua 106: 81–112.

[7] MacWhinney B (1978) The Acquisition of Morphophonology. Monographs of the Society For Research In Child Development 43: 1–122. 752799

[8] Pine JM, Lieven EVM, Rowland CF (1998) Comparing different models of the development of the English verb category. Linguistics 36: 807–830.

[9] Tomasello M (2000) The item-based nature of children’s early syntactic development. Trends in Cognitive Sciences 4: 156–163. 10740280 10.1016/s1364-6613(00)01462-5

[10] Wexler K (1998) Very early parameter setting and the unique checking constraint: A new explanation of the optional infinitive stage. Lingua 106: 23–79.

[11] Wilson S (2003) Lexically specific constructions in the acquisition of inflection in English. Journal of Child Language 30: 71–115. 10.1017/s0305000902005512 12718294

[12] Pizzuto E, Caselli MC (1992) The acquisition of Italian morphology: implications for models of language development. Journal of Child Language 19: 491–557. 1429947 10.1017/s0305000900011557

[13] Yang C (2013) Ontogeny and phylogeny of language. Proceedings of the National Academy of Sciences 110: 6324–6327. 10.1073/pnas.1216803110 23576720 PMC3631656

[14] Zipf GK (1949) Human behavior and the principle of least effort: An introduction to human ecology Oxford: Addison-Wesley.

[15] Rubino RB, Pine JM (1998) Subject–verb agreement in Brazilian Portuguese: what low error rates hide. Journal of Child Language 25: 35–39. 9604568 10.1017/s0305000997003310

[16] Pinker S (1984) Language learnability and language development Cambridge, MA: Harvard University Press.

[17] Deen KU (2005) Productive agreement in Swahili: Against a piecemeal approach In: Brugos A, Clark-Cotton MR, Ha S, editors. Proceedings of the 29th Annual Boston University Conference on Language Development Boston: Cascadilla Press pp. 156–167.

[18] Legate JA, Yang C (2007) Morphosyntactic learning and the development of tense. Language Acquisition 14: 315–344.

[19] Blom E, Wijnen F (2013) Optionality of finiteness: Evidence for a no-overlap stage in Dutch child language. First Language 33: 225–245.

[20] Gathercole VCM, Sebastián E, Soto P (1999) The early acquisition of Spanish verbal morphology: Across-the-board or piecemeal knowledge? The International Journal of Bilinguialism 3: 133–182.

[21] Harris T, Wexler K (1996) The optional infinitive stage in child English: Evidence from negation In: Clahsen H, editor. Generative approaches to first and second language acquisition Amsterdam: Benjamins pp. 1–42.

[22] Clahsen H, Eisenbaiss S, Penke M (1996) Lexical Learning in Early Syntactic Development In: Clahsen H, editor. Generative Perspectives on Language Acquisition. Amsterdam: John Benjamins pp. 129–159.

[23] Hyams N (1994) Nondiscreteness and Variation in Child Language: Implications for Principle and Parameter Models of Language Development In: Levy Y, editor. Other Children, Other Languages: Issues in the Theory of Language Acquisition. Hillsdale; NJ: Lawrence Erlbaum Associates pp. 11–40.

[24] Pine JM, Freudenthal D, Krajewski G, Gobet F (2013) Do young children have adult-like syntactic categories? Zipf’s law and the case of the determiner. Cognition 127: 345–360. 10.1016/j.cognition.2013.02.006 23542410

[25] Cipriani P, Chilosi AM, Bottari P, Pfanner L (1991) L’acquisizione della morfosintassi: Fasi e processi Padova: Unipress.

[26] Serra M, Solé R (1992) Language acquisition in Spanish and Catalan children: a longitudinal study. Barcelona: Universitat de Barcelona.

[27] Maratsos M (2000) More overgeneralizations after all: new data and discussion on Marcus, Pinker, Ulman, Hollander, Rosen & Xu. Journal of Child Language 27: 183–212. 10740972 10.1017/s0305000999004067

[28] Akhtar N, Tomasello M (1997) Young children's productivity with word order and verb morphology. Developmental Psychology 33: 952–965. 9383618 10.1037//0012-1649.33.6.952

[29] Olguin R, Tomasello M (1993) Twenty-five-month-old children do not have a grammatical category of verb. Cognitive Development 8: 245–272.

[30] Theakston AL, Lieven EVM, Pine JM, Rowland CF (2005) The acquisition of auxiliary syntax: BE and HAVE. Cognitive Linguistics 16: 247–277.

[31] Alcoba S (1999) La Flexión Verbal In: Bosque I, Demonte V, editors. Gramática Descriptiva de la Lengua Española. Madrid: Espasa-Calpe pp. 4915–4991.

[32] Aguado-Orea J (2004) The acquisition of morpho-syntax in Spanish: Implications for current theories of development [PhD Thesis]. Nottingham: University of Nottingham. 393 p.

[33] López Ornat S, Fernández A, Gallo P, Mariscal S (1994) La adquisición de la lengua española Madrid: Siglo XXI.

[34] Gathercole VCM, Sebastián E, Soto P (2002) The Emergence of Linguistic Person in Spanish-Speaking Children. Language Learning 52: 679–722.

[35] MacWhinney B (2000) The CHILDES Project: Tools for Analyzing Talk. Mahwah, NJ: Lawrence Erlbaum Associates.

[36] Jackson-Maldonado D, Thal D, Marchman VA, Bates E, Gutierrez-Clellen V (1993) Early lexical development in Spanish-speaking infants and toddlers. Journal of Child Language 20(3): 523–549. 8300774 10.1017/s0305000900008461

[37] Serra M, Serrat E, Solé R, Bel A, Aparici M (2000) La adquisición del lenguaje Barcelona: Ariel.

[38] Barton ME, Tomasello M (1994) The rest of the family: the role of fathers and siblings in early language development In: Gallaway C, Richards BJ, editors. Input and Interaction in Language Acquisition. Cambridge: Cambridge University Press pp. 107–134.

[39] Perez-Pereira M (1989) The acquisition of morphemes: Some evidence from Spanish. Journal of Psycholinguistic Research 18: 289–312. 2746554 10.1007/BF01067038

[40] Radford A, Ploennig-Pacheco I (1995) The morpho-syntax of subjects and verbs in child Spanish: A case study. Essex Reports in Linguistics 5: 23–67.

[41] Grinstead J, De la Mora J, Vega-Mendoza M, Flores B (2009) An elicited production test of the Optional Infinitive stage in Child Spanish In: Crawford J, Otaki K, Takahashi M, editors. Proceedings of the 3rd Conference on Generative approaches to Language Acqusition North America. Somerville: Cascadilla Press pp. 36–45.

[42] Räsänen SHM, Ambridge B, Pine JM (2014) Infinitives or bare stems? Are English-speaking children defaulting to the highest frequency form?. Journal of Child Language 41: 756–779. 10.1017/S0305000913000159 23830201

